# Functional Iron Deficiency in Patients With Heart Failure: A Focus on Its Prevalence Among Hospitalized Patients and Short-Term Outcomes

**DOI:** 10.7759/cureus.92074

**Published:** 2025-09-11

**Authors:** Karthik Gopinath, Ravi Sankar Tulluru, Abraham Speedie

**Affiliations:** 1 Cardiology, Christian Medical College, Vellore, IND

**Keywords:** anemia, functional iron deficiency, heart failure, prevalence, south india

## Abstract

Background

Iron deficiency (ID) is common in heart failure (HF) and is present in up to 80% of hospitalized patients. The majority of the studies have focused on describing patients with low ferritin levels. We studied functional iron deficiency (FID) in heart failure in a single-center observational study, with a focus on its relevance in the current era and its impact on outcomes in hospitalised patients.

Methods

Patients with HF and normal hemoglobin were considered and divided into 2 groups: absolute ID (serum ferritin < 100 mg/L) and FID (normal serum ferritin, i.e., 100-300 mg/L with low transferrin saturation (TSAT; <20%). Anemia was defined as hemoglobin (Hb) <13 g/dl for males and <12 g/dl for females.

Patients were classified into four HF classes based on ejection fraction (EF). EF of ≥50% was taken as HFpEF (heart failure with preserved ejection fraction), 41-49% EF as HFmrEF (heart failure with mildly reduced ejection fraction), and EF <40% as HFrEF (heart failure with reduced ejection fraction). A one-year follow-up was done to assess the New York Heart Association (NYHA) functional class, hemoglobin levels, and recurrent hospitalizations (if any).

Results

In total, 53 patients, of whom 43 (81%) were males and 10 (19%) were females, were studied. Follow-up data of 29 patients showed outcomes such as deaths (n=6; 11%), recurrent hospitalization for HF (8 patients; 24%), and prolonged hospital stay (n=1). Twenty-four patients (45%) showed clinical improvement on follow-up, among whom 15 patients (62.5%) received IV iron supplementation.

Conclusion

Functional ID, as determined by low TSAT, represents a significant proportion of HF who are iron-deficient. These patients have outcomes similar to those of patients with normal iron levels. IV iron supplementation resulted in symptomatic improvement; however, this was not statistically significant. In view of this, we hypothesize that the term "functional iron deficiency" may not be deemed relevant in the context of HF, especially in the presence of multiple other factors influencing iron levels. Further large-scale studies are needed to establish this.

## Introduction

Iron deficiency (ID) is the most common nutritional disorder, affecting over one-third of the global population [1]. It is especially prevalent among individuals with heart failure (HF), affecting 55% of chronic and up to 80% of acute HF patients, regardless of ejection fraction (EF) [2,3]. Furthermore, ID is an independent predictor of poorer functional capacity and reduced survival rates [4-7].

ID has typically been considered primarily in the context of anemia, particularly in patients with chronic heart failure (CHF) [8,9]. As a result, the prevalence of ID has, in most cases, been documented in CHF patients who also have anaemia.

Research indicates that most iron-deficient patients exhibit functional ID (FID), characterized by serum ferritin levels between 100 and 300 mg/L and transferrin saturation (TSAT) below 20%. This condition arises from impaired iron metabolism due to the inflammatory processes associated with CHF [10]. FID is the main cause of low iron levels in patients with CHF [10]. However, multiple other mechanisms operate in HF, and this complex interplay contributes to the development of FID. Chronic kidney disease (CKD) contributes to anemia through several known mechanisms [11]. Long-term therapy with angiotensin-converting enzyme (ACE) inhibitors and angiotensin receptor blockers (ARBs) causes a modest reduction of serum hemoglobin levels by decreasing the production of erythroid progenitors and by inhibiting hematopoiesis [12-14]. Anorexia contributes to overt or absolute ID [15].

The primary objective of this study was to estimate the prevalence of FID among patients with HF and ID, admitted to our HF unit. A secondary objective was to assess whether patients with FID differed from those with normal iron profiles in terms of baseline clinical and echocardiographic characteristics. Finally, we evaluated follow-up outcomes, including changes in New York Heart Association (NYHA) functional class, serial hemoglobin levels, and major adverse cardiovascular events (MACEs).

## Materials and methods

This study is a single-center retrospective analysis conducted at a large tertiary care hospital in Vellore, South India, from January 2020 to July 2022. We examined 284 patients who were admitted to our HF unit with a clinical diagnosis of HF during this period. Data were extracted from inpatient electronic health records, discharge summaries, and laboratory databases. Data collection was performed by Authors 1 and 2, with Author 3 supervising and resolving any disagreements by consensus. The diagnosis of HF was determined in accordance with the 2022 American Heart Association (AHA) guidelines. Anemia was defined as hemoglobin (Hb) <13 g/dl for males and <12 g/dl for females [16]. As per guidelines, ID was defined as a ferritin <100 ng/mL or a ferritin 100 to 299 ng/mL if transferrin saturation (TSAT) is <20% [17,18]. We included only patients with serum ferritin levels greater than 100 mcg/L and transferrin saturation levels below 20%, i.e., FID [19]. Patients with absolute ID, defined as serum ferritin levels below 100 mcg/L, were excluded from the study. Major adverse cardiovascular events (MACE) were defined as nonfatal stroke, nonfatal myocardial infarction, or cardiovascular death. Additionally, unexpected prolonged hospitalization (>14 days) related to HF was included in the composite endpoint. Clinical improvement was defined as improvement of ≥1 NYHA functional class with no HF hospitalization or death during follow-up; the six-minute walk test was not performed. Rehospitalization was defined as any unplanned admission for worsening HF.

This study aimed to estimate the prevalence, spectrum, relevance, and clinical outcomes (NYHA functional class at follow-up and the occurrence of MACE) of HF patients diagnosed with FID during their initial hospitalization. Hospitalizations and deaths were verified using discharge summaries and electronic hospital records. The cause of death was adjudicated by a review of available records.

Data from male and female patients over 18 years of age who were clinically diagnosed with HF were analyzed. Patients with other clear causes of ID, such as anemia due to hemorrhoids or malignancy, were excluded. Patients with missing baseline iron studies were excluded (n=7). For other variables, if <5% of data were missing, a complete case analysis was performed. No imputation was used. A diagrammatic algorithm of patient selection is shown below.

All relevant details were extracted from the patient charts, including dietary history, clinical findings at presentation, and the presence of comorbidities such as diabetes mellitus, systemic hypertension, chronic kidney disease (CKD), and obstructive sleep apnea. Additionally, findings from ECG and transthoracic echocardiography were noted.

The data were further analyzed to identify patients with persistent low hemoglobin levels, their NYHA functional class upon follow-up, and their history of recurrent hospitalizations. Patients were classified into different categories of HF based on their EF. Those with an EF of ≥50% were classified as HFpEF (heart failure with preserved ejection fraction), those with an EF of 41-49% as HFmrEF (heart failure with mildly reduced ejection fraction), those with an EF of ≤40% as HFrEF (heart failure with reduced ejection fraction), and those with an EF of ≤30% as having severe left ventricular dysfunction.

In addition to routine hemogram data, the patients' iron status was assessed through an analysis of serum iron, serum ferritin, total iron binding capacity, and TSAT. Other laboratory parameters investigated included N-terminal B-type natriuretic peptide (NT-ProBNP), thyroid-stimulating hormone (TSH) levels, and chest X-ray findings. None of our patients was on long-term valsartan therapy.

Anemia was defined according to the World Health Organization's criteria as hemoglobin (Hb) levels below 13 g/dL for males and below 12 g/dL for females [16]. Absolute ID was identified with serum ferritin levels below 100 mg/L, while functional ID was characterized by normal serum ferritin levels (between 100 and 300 mg/L) combined with low TSAT levels (below 20%). All patients received guideline-directed medical therapy (GDMT) as per the physician's discretion. Data were recorded on the use and dose of ACE inhibitors (ACEis)/ARBs/angiotensin receptor-neprilysin inhibitors (ARNIs), β-blockers, mineralocorticoid receptor antagonists (MRAs), and sodium-glucose cotransporter 2 (SGLT2) inhibitors, including whether ≥50% of the target guideline dose was achieved (per European Society of Cardiology (ESC) guidelines) [18]. IV iron therapy (ferric carboxymaltose) was administered selectively in patients with ID at the discretion of the treating physician.

**Figure 1:**
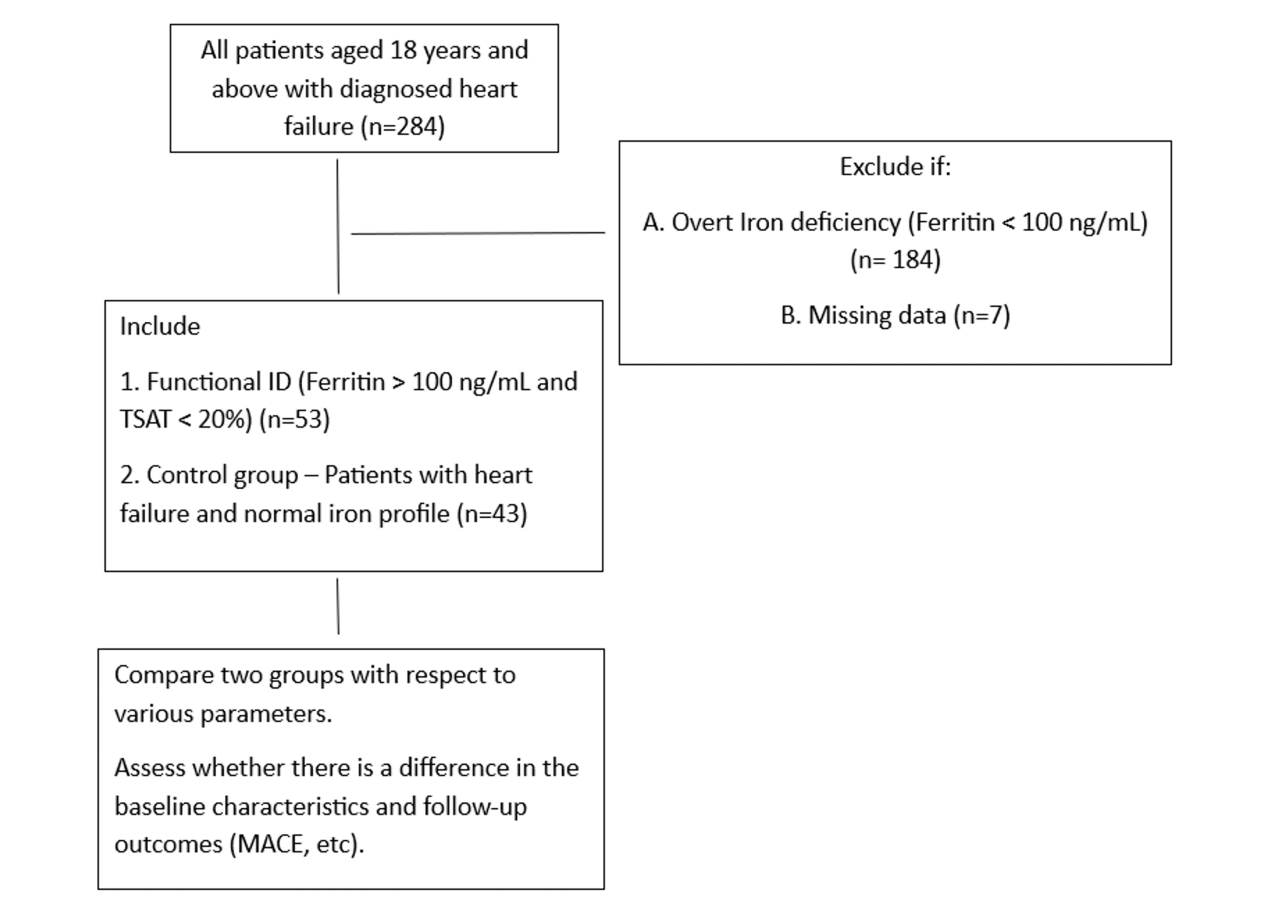
Diagrammatic algorithm of the study

Figure 1 shows the diagrammatic algorithm of the study.

Statistical methods

Descriptive and inferential statistical analyses have been carried out in the present study. Results on continuous measurements are presented as Mean ± SD (Min-Max), and results on categorical measurements are presented as Number (%). Significance is assessed at a 5% level of significance. The following assumptions on data are made: 1. Dependent variables should be normally distributed, 2. Samples were drawn from the population.

The chi-square/Fisher's Exact test has been used to find the significance of study parameters on a categorical scale between two or more groups, in a non-parametric setting for qualitative data analysis. The Fisher's Exact test is used when cell samples are very small.

The student's t-test (two-tailed, independent) has been used to find the significance of study parameters on a continuous scale between two groups (Inter-group analysis) on metric parameters. Levene's test for homogeneity of variance has been performed to assess the homogeneity of variance. A t-test is a statistical test that is used to compare the means of two groups. It is often used in hypothesis testing to determine whether a process or treatment has an effect on the population of interest, or whether two groups are different from one another. The null hypothesis (H0) is that the true difference between these groups' means is zero, and the alternative hypothesis (Ha) is that the true difference is different from zero.

Statistical software

SPSS 22.0 (IBM Corp., Armonk, NY, US) and the R environment ver.3.2.2 (R Core Team (2021). R: A language and environment for statistical computing. R Foundation for Statistical Computing, Vienna, Austria) were used for the analysis of the data, and Microsoft Word and Excel (Microsoft Corporation, Redmond, WA, US) were used to generate graphs, tables, etc.

## Results

Data from 53 patients with FID were analyzed. Of these patients, 43 (81%) were male and 10 (19%) were female. The mean age of the participants was 57.3 years (± 13.03 years). Most patients presented with NYHA functional class III (39.6%, n=21), and the mean EF was 35%, indicating the subclass of HFrEF. 

Comorbidities identified in the study included diabetes mellitus in 35 (66%) patients, systemic hypertension in 26 patients (49.1%), and CKD in 10 patients (18.9%). The majority of the patients, i.e., 47 (88%), presented with dyspnea as their chief complaint. Out of 53 patients, 23 (43.4%) exhibited acute pulmonary edema, classified as NYHA Class IV HF, while 12 patients (22%) presented with acute coronary syndrome (ACS). Atrial fibrillation was diagnosed in 3 patients, and 13 (24%) had displayed a wide QRS complex on their electrocardiograms.

The baseline echocardiographic parameters indicated that 17 patients (33% ) had significant mitral regurgitation (MR), and 9 patients (17%) had coexisting valvular heart disease, primarily stenotic lesions.

Hemoglobin and iron indices were reported as follows: the mean hemoglobin level was 11.1 ± 1.75 g/dL, the mean ferritin level was 278.19 mcg/dL (± 254.63), and the mean transferrin saturation was 12%. Additionally, the mean NT-proBNP level was 8855.26 ± 11225.63 ng/L. specifically. Forty-one patients (77%) presented with HFrEF, defined as an EF of ≤ 40%. Among these, 29 (54.74%) exhibited severe left ventricular dysfunction, characterized by an EF of ≤ 30%.

In our study, 45 participants (84%) had anemia, while the remainder did not. The type of anemia observed was predominantly microcytic hypochromic. Thirty-one (72%) of those without ID had anemia. Among the study population, 5 patients (9.4%) with FID and four patients (9.3%) with normal iron profile required blood transfusion, with no significant difference between groups (chi-square, p = 1.000). Similarly, intravenous iron therapy (ferric carboxymaltose) was administered in 25 patients (47.2%) with FID compared to 23 patients (53.5%) in the control group, which was not statistically significant (chi-square, p = 0.681). Gastroscopy was performed in one patient with FID versus five patients (11.6%) with normal iron profile, again without a significant difference (chi-square, p = 0.124).

As shown in Table 1, we compared patients with FID to those with normal iron levels. Although adverse outcomes, such as recurrent hospitalization, mortality, and prolonged hospital stays, were more common in patients with FID, these differences were not statistically significant. Other factors, including the incidence of various presentations of HF, NYHA class at presentation, and duration of hospitalization, were similar between the two groups. Notably, NT-proBNP levels were lower in the group with FID.

**Table 1:** Comparison of baseline and outcome parameters between patients with functional iron deficiency and those with normal iron levels Values are presented as mean ± standard deviation (SD) for continuous variables and n (%) for categorical variables. Continuous variables were compared using the independent samples t-test (or Mann–Whitney U test for non-normally distributed data). Categorical variables were compared using the Chi-square or Fisher’s exact test, as appropriate. The data represent the mean hemoglobin values of the study cohort, which included both anemic and non-anemic patients. Comparison of baseline and outcome characteristics between functional iron deficiency and normal iron groups showed no significant differences in most demographic, clinical, echocardiographic, or laboratory parameters. Notable exceptions were a lower transferrin saturation and a higher prevalence of mitral regurgitation in the functional iron deficiency group. These findings suggest broad clinical similarity between the two cohorts, with iron indices being the main differentiating factor. Hb: hemoglobin; TIBC: total iron-binding capacity; NT-proBNP: N-terminal pro–B-type natriuretic peptide; TSH: thyroid-stimulating hormone; MR: mitral regurgitation; LVSD: left ventricular systolic dysfunction; HFpEF: heart failure with preserved ejection fraction; HFmrEF: heart failure with mildly reduced ejection fraction; HFrEF: heart failure with reduced ejection fraction; MACE: major adverse cardiovascular events; ACS: acute coronary syndrome; CKD: chronic kidney disease; NYHA: New York Heart Association

	Functional iron deficiency	Normal iron levels	Statistical test used	P value	
Study parameter	Characteristics of study population (Mean)	Characteristics of study population (Mean)			
Total	53	43			
Age groups	Mean age 57.35±13.03 yrs <18 yrs: None ≥ 65 yrs: 13	Mean age 57.91 ± 11.54 yrs <18 yrs: None ≥ 65 yrs: 11	t-test	0.824	
Males	43 (81.1%)	33 (76.7%)	Chi-square	0.784	
Females	10 (18.9%)	10 (23.3%)	Chi-square	0.784	
Angina as the chief complaint	14 (26.4%%)	6 (14.0%)	Chi-square	0.214	
Dyspnea as the chief complaint	47 (88.7%)	36 (83.7%)	Chi-square	0.685	
ACS at presentation	12 (22.6%)	3 (7.0%)	Fisher’s exact	0.048	
Hypertension	26 (49.1%)	23 (53.5%)	Chi-square	0.821	
Diabetes mellitus	35 (66.0%)	27 (62.8%)	Chi-square	0.907	
Chronic kidney disease (CKD)	10 (18.9%)	14 (32.6%)	Chi-square	0.192	
NYHA class at presentation	Total (percentage)	Total (percentage)			
NYHA class 2	10 (18.9%)	6 (14.0%)	Chi-square	0.714	
NYHA class 3	21 (39.6%)	23 (53.5%)	Chi-square	0.250	
NYHA Class 4	23 (43.4%)	14 (32.6%)	Chi-square	0.382	
HFpEF	5 (9.4%)	5 (11.6%)	Fisher’s exact	0.989	
HFmrEF	6 (11.3%)	2 (4.7%)	Fisher’s exact	0.290	
HFrEF	41 (77.4%)	36 (83.7%)	Chi-square	0.603	
Echocardiographic parameters					
Severe LVSD	29 (54.7%)	24 (55.8%)	Chi-square	1.0	
Significant mitral regurgitation (Moderate and Severe MR)	17 (32.1%)	0 (0.0%)	Fisher’s exact	<0.001	
Co-existing valvular heart disease	9 (17.0%)	2 (4.7%)	Fisher’s exact	0.104	
ECG parameters					
Atrial fibrillation	3 (5.7%)	3 (7.0%)	Fisher’s exact test	1.0	
Wide QRS	13 (24.5%)	9 (20.9%)	Fisher’s exact test	0.80	
Laboratory parameters					
Mean hemoglobin (g/dl)	11.1±1.75	11.27 ± 2.16	Independent t-test (Welch)	0.678	
Mean serum iron (mcg/dl)	33.0 ± 11.97	75.93 ± 29.00	Independent t-test (Welch)	<0.001	
Mean serum ferritin (mcg/L)	278.19 ± 254.63	413.31 ± 567.03	Independent t-test (Welch)	0.153	
TIBC (mcmol/L)	274 ± 69.47	260.51 ± 79.81	Independent t-test (Welch)	0.386	
Transferrin saturation	12% ± 5%	31% ± 13%	Independent samples t-test	<0.001	
Creatinine (mg/dl)	1.98 + 2.48	1.71 ± 1.91	Welch’s t-test	0.55	
NT-ProBNP (ng/L)	8855.26±11225.63	13136.58 ± 11850.14	Welch’s t-test	0.075	
TSH (mU/L)	3.95±8.14	3.76 ± 3.52	Welch’s t-test	0.88	
CXR showing consolidation	15 (28.3%)	2 (4.7%)	Chi-square	0.006	
MACE	(Among those who followed up: n = 41) Total n=15 (36.5%)	(Among those who followed up: n = 37) Total n=9 (24.3%)	Chi-square	0.354	
Deaths	7 (13.2%)	2 (5.4%)	Chi-square	0.281	
Prolonged hospitalization	1 (1.9%)	None	Chi-square	1.000	
Recurrent hospitalization for heart failure	10 (18.9%)	7 (18.9%)	Chi-square	0.951	

Baseline pharmacological therapy was comparable between patients with FID and those with a normal iron profile (Table 2). The proportion receiving ACEI/ARB/ARNI (42.2% vs. 40.0%, p=1.000) and β-blockers (62.2% vs. 60.0%, p=1.000) did not differ significantly between groups. Similarly, the use of mineralocorticoid receptor antagonists (44.4% vs. 37.5%, p=0.668) and the attainment of ≥50% target doses of ACEI/ARB/ARNI or β-blockers showed no significant variation. Rates of SGLT2 inhibitor prescription, antiplatelet and anticoagulant therapy, and recent iron or ESA use were also similar across groups. These findings suggest that differences in outcomes are unlikely to be explained by background medical therapy.

**Table 2:** Baseline guideline-directed medical therapy and adjunctive treatments in patients with functional iron deficiency versus normal iron profile, including the proportion achieving ≥50% of target doses Medication classes shown include angiotensin-converting enzyme inhibitors (ACEi), angiotensin receptor blockers (ARB), angiotensin receptor–neprilysin inhibitor (ARNI), β-blockers, mineralocorticoid receptor antagonists (MRA), and sodium–glucose cotransporter 2 inhibitors (SGLT2i). Loop diuretic doses are reported in furosemide equivalents (FEQ) (conversion: furosemide 40 mg = torsemide 20 mg). Antiplatelet agents were prescribed for patients with coronary or peripheral arterial disease; anticoagulants were prescribed for atrial fibrillation or venous thromboembolism. “≥50% target dose” is defined as the achievement of at least half of the guideline-directed target dose as per European Society of Cardiology (ESC) recommendations. Iron therapy refers to oral or intravenous iron administration within the preceding three months; erythropoiesis-stimulating agent (ESA) use is also reported. Across both groups, no significant differences were observed in background guideline-directed medical therapy (GDMT), iron therapy, or ESA use, suggesting medications are unlikely to explain outcome differences.

Medication/Therapy	Functional ID (n=53)	Normal Iron (n=43)	p-value
ACEI/ARB/ARNI, n (%)	19 (42.2%)	16 (40.0%)	1.000
≥50% target ACEI/ARB/ARNI dose, n (%)	6 (30.0%)	2 (12.5%)	0.257
β-blocker, n (%)	28 (62.2%)	24 (60.0%)	1.000
≥50% target β-blocker dose, n (%)	3 (9.7%)	4 (16.7%)	0.686
MRA, n (%)	20 (44.4%)	15 (37.5%)	0.668
≥50% target MRA dose, n (%)	20 (90.9%)	9 (60.0%)	0.042
SGLT2 inhibitor, n (%)	8 (17.8%)	6 (15.0%)	0.959
Antiplatelet, n (%)	27 (58.7%)	22 (55.0%)	0.899
Anticoagulant, n (%)	2 (4.3%)	2 (5.0%)	1.000
IV iron within last 3 months, n (%)	0 (0.0%)	1 (2.5%)	0.471
Oral iron within the last 3 months, n (%)	0 (0.0%)	3 (7.5%)	0.097
ESA use within the last 3 months, n (%)	0 (0.0%)	1 (2.5%)	0.465

The short-term outcomes of the study participants were analyzed. Out of 53 patients, 16 (30%) did not follow up. Among the remaining 37 patients, major adverse cardiac events during the 6-month follow-up included 6 deaths (11%), with 7 of them (24%) having hospitalizations for recurrent HF. Of these seven patients, three received IV iron during their initial hospitalization, and one had a prolonged hospital stay. Additionally, 22 patients (75%) experienced symptomatic improvement in their NYHA class on follow-up in response to guideline-directed therapy. Notably, 15 patients (68%) of those who showed improvement received IV iron supplementation during their initial hospitalization.

## Discussion

The findings of this study emphasize the significant number of patients with HF who have FID, characterized by normal ferritin levels and low transferrin saturation. These patients may be overlooked if only serum ferritin is used to assess iron levels without considering transferrin.

ID is widely prevalent in HF, affecting over 50% of ambulatory patients, according to American studies [2,3], and prevalence rates between 60% and 88% as per Indian studies [20-22]. Previous studies in India have reported a prevalence of 60% among hospitalized patients. Most of these studies have focused on overall ID, which includes both absolute and FID. Specifically, the prevalence of FID alone has been reported as 28% in earlier studies [23]. In our study, we found that 53 patients, accounting for 19%, had FID.

The majority of patients with FID were males (81%), which is atypical compared to previous HF studies, where females are predominantly affected. Additionally, being female has been reported as a risk factor for developing HF, especially in the context of low iron levels [24].

Anemia was present in the majority of our patients (84%). Among those who were readmitted for HF, 62% had lower hemoglobin levels as compared to their hemoglobin levels during the initial hospitalization. A prior study by Yeo Tee et al. indicated that anemia was unrelated to the underlying functional iron-deficient state [25].

ID is an independent risk factor for adverse outcomes in HF, regardless of serum hemoglobin levels. In our study, we classified patients based on TSAT, and a significant proportion of these patients experienced poor outcomes.

HF is characterized by a systemic inflammatory state, which leads to elevated levels of hepcidin. This disproportionate increase in serum hepcidin serves as a molecular hallmark of a functional iron-deficient state [26]. Under normal circumstances, hepcidin inhibits the release of iron from the reticuloendothelial system (RES) when body iron levels are adequate. However, in the context of HF, ongoing low-grade inflammation results in an excessive increase in hepcidin, combined with low iron levels from various causes (‘iron adaptation’) [27]. This combination ultimately leads to ID [28]. In addition to the chronic inflammatory state, a combination of multiple other factors contributes to the low iron levels as mentioned previously [29].

This study has several limitations, including its retrospective design, small sample size, and limited follow-up, with nearly one-third of patients lost to follow-up, reducing statistical power and introducing potential attrition bias. Most FID patients also had anemia, but subgroup analysis by anemia status was not feasible due to small numbers. IV iron therapy was administered at the physician's discretion without a uniform protocol, creating treatment bias, and our analysis relied only on univariate comparisons without multivariate adjustment for key confounders such as anemia, CKD, and HF severity. As a result, our findings, though highlighting the prevalence of FID in HF, should be interpreted with caution and regarded as exploratory and hypothesis-generating rather than definitive evidence regarding its prognostic significance.

## Conclusions

In this cohort, no significant differences were observed between patients with functional iron deficiency and those with normal iron profiles. Given the retrospective design, small sample size, incomplete follow-up, and lack of multivariate adjustment, these findings should be regarded as exploratory and hypothesis-generating. Our study suggests that functional iron deficiency, while common in heart failure, may have limited clinical relevance in this context, with outcomes comparable to those of patients with normal iron levels. We hypothesize that functional iron deficiency could represent an epiphenomenon, reflecting the broader impact of chronic disease and inflammation rather than an independent prognostic factor. Further large-scale, prospective studies are warranted to clarify its role and true clinical significance in heart failure populations.
